# Compounds from the Roots and Rhizomes of *Valeriana amurensis *Protect against Neurotoxicity in PC12 Cells

**DOI:** 10.3390/molecules171215013

**Published:** 2012-12-18

**Authors:** Qiuhong Wang, Changfu Wang, Yueming Zuo, Zhibin Wang, Bingyou Yang, Haixue Kuang

**Affiliations:** 1 Key Laboratory of Chinese Materia Medica (Ministry of Education), Heilongjiang University of Chinese Medicine, No. 24 HePing Road, XiangFang District, Harbin 150040, China; E-Mails: qhwang668@sina.com (Q.W.); wangchangfu831124@163.com (C.W.); wzbmailbox@126.com (Z.W.); ybywater@163.com (B.Y.); 2 Jiangxi University of Traditional Chinese Medicine, Nanchang 330006, China; E-Mail: zuo_yueming@163.com

**Keywords:** *Valeriana amurensis*, germacrane-type sesquiterpenoids, heishuixiecaoline, lignans, PC 12 cell

## Abstract

Three new germacrane-type sesquiterpenoids, heishuixiecaoline A–C (compounds **1**–**3**), were isolated along with ten known compounds **4**–**13** from fraction of *Valeriana amurensis* roots and rhizomes effective against Alzheimer’s disease (AD). The structures of **1**–**3** were elucidated on the basis of their spectroscopic data. We also investigated the protective effect of compounds **1**–**13** on the neurotoxicity of PC12 cells induced by amyloid-beta (Aβ_25–25_), respectively. As a result, germacrane-type sesquiterpenoids **1**–**4** and lignans **5**–**7** were seen to afford protection against Aβ-induced toxicity in PC 12 cells. This study will contribute to revealing the chemical basis for the therapeutic effect of *V. amurensis* against AD.

## 1. Introduction

The genus *Valeriana* has been taxonomically placed in the family *Valerianaceae*, comprising *ca.* 30 species in China [1]. *Valerian* (*Valerianacae*) is a perennial herb and some species in this genus have been used widely as a mild sedative and sleep aid for centuries in Europe and North America [2,3,4]. Previous studies on genus *Valeriana* plants indicated anxiolytic, antidepressant, antispasmodic, sedative, antitumor, and anti-HIV activities [5,6,7,8,9]. As one species of *Valeriana*, *Valeriana amurensis* is abundantly distributed in northeast China, especially in the Great Xing’an Mountains area. However, there were no investigations about its pharmacology activity and chemical constituents except for the sedative and anti-hyperspasmia effects of *V. amurensis* volatile oil [10] until we reported its potential therapeutic effect in Alzheimer’s disease (AD) for the first time [11,12]. We have also screened and determined the AD-effective fraction (50% EtOH fraction from AB-8 macroporous resin column of 95% EtOH extract) from the previous studies [11,12], based on which a bioassay-guided isolation and phytochemical study of *V. amurensis* was performed and three new and ten known compounds were obtained from the effective fraction. The structures of known compounds **4**–**13** were determined by detailed 1D- and 2D-NMR analyses, ESI-MS and comparison of their spectral data with literature values. In this paper, the isolation and structural elucidation of the new germacrane-type sesquiterpenoids **1**–**3** is described. We also investigated the neuroprotective effects of compounds **1**–**15** in a PC12 neuronal cell line. The PC12 cell line, derived from rat pheochromocytoma, displays phenotypic characteristics of sympathetic neurons. The cells were grown in the presence of various toxins mimicking the conditions taking place in neurodegenerative diseases, including amyloid-beta (Aβ, the peptide composing the amyloid plaques in brains of AD patients).

## 2. Results and Discussion

Compound **1** was obtained as white amorphous powder, and assigned the molecular formula C_17_H_24_O_3_ from its HRESIMS (*m*/*z* 299.1619 [M+Na]^+^, calc. for C_17_H_24_O_3_Na, 299.1623) and NMR data (Table 1). Six degrees of unsaturation can be concluded for **1** according to the molecular formula C_17_H_24_O_3_. The IR spectrum displayed the presence of carbonyl (1736 cm^−1^), *α*,*β*-unsaturated aldehyde (1682 cm^−1^), and double-bond (1625 cm^−1^) absorptions.

**Figure 1:**
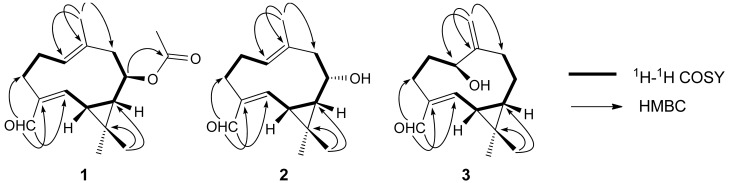
Key ^1^H-^1^H COSY and HMBC correlations of compounds **1**–**3**.

The ^1^H-NMR spectrum of compound **1** (Table 1) displayed four methyl singlets [*δ*_H_ 1.18 (H-12), 1.20 (H-13), 1.34 (H-15), 2.03 (H-17)], signals for two olefinic protons [*δ*_H_ 5.31 (H-1), 6.57 (H-5)], and an oxygenated methine proton [*δ*_H_ 4.50 (H-8)], and an aldehydic proton [*δ*_H_ 9.30 (H-14)]. Analysis of its ^13^C-NMR and DEPT spectra showed 17 carbon resonances, including four methyls, three methylenes, three methines (one oxygenated), two trisubstituted double bond [*δ*_C_ 145.1 (C-4), 155.8 (C-5), 128.7(C-1), 133.7 (C-10)], a carbonyl carbon [*δ*_C_ 172.1 (C-16)], and an aldehydic carbon [*δ*_C_ 196.6 (C-14)]. The acetate group was determined to be located at C-8 by the HMBC correlations from H-8 (*δ*_H_ 4.50) to C-16, C-11 (*δ*_C_ 23.7), C-7 (*δ*_C_ 40.8), and C-9 (*δ*_C_ 47.2). The other correlations in the HMBC and ^1^H-^1^H-COSY spectra as shown in Figure 1 confirmed the connectivities in compound **1**.

**Figure 2:**
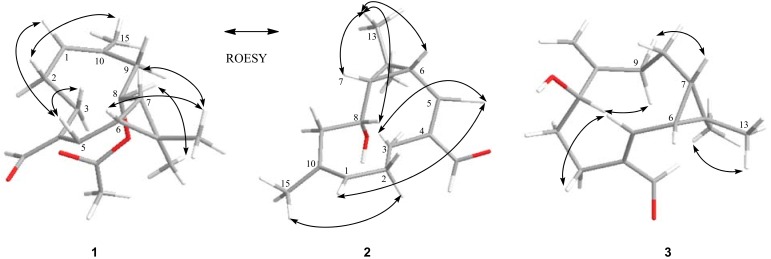
Key ROESY correlations of compounds **1**–**3**.

The relative configurations at C-6, C-7, and C-8 in **1** were deduced by a ROESY experiment (Figure 2) and ^1^H-NMR coupling constants [13]. 

**Figure 3:**
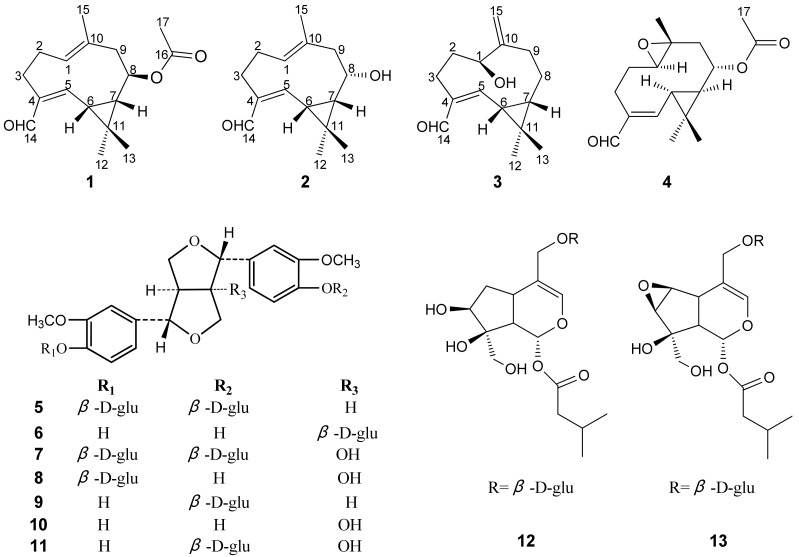
Structures of compounds **1**–**13**.

The coupling constant of 9.8 Hz and the NOE correlations between H-6 and H-7 suggested *syn* configuration of the cyclopropane moiety, the *β*-orientation of H-6 and H-7 were assigned by the correlations of H-7/CH_3_-13 and H-6/CH_3_-13. The *α*-orientation of H-8 was established by the correlations of H-8/CH_3_-12. The correlations of H-5/H-3a and H-2 (a, b)/CH_3_-15 indicated Δ^4,5^ and Δ^1,10^ to be *Z*- and *E*-configured, respectively, which was confirmed by a key NOE correlation of H-5 with H-1. Therefore, the structure of compound **1** was established as 10-methyl-6,7-dimethyl-methylene-8*β*-acetoxy-4-aldehyde-(4*Z*,10*E*)-dicyclodecadiene (Figure 3), and this compound was named heishuixiecaoline A.

Compound **2** was isolated as white amorphous powder, and its molecular formula was determined to be C_15_H_22_O_2_ by HRESIMS (*m*/*z* 257.1514 [M+Na]^+^, calc. for C_15_H_22_O_2_Na, 257.1517), requiring five degrees of unsaturation. The IR spectrum displayed the presence of hydroxyl (3429 cm^−1^), *α*,*β*-unsaturated aldehyde (1689 cm^−1^), and double-bond (1624 cm^−1^) absorptions. The ^1^H- and ^13^C-NMR spectra data (Table 1 and Table 2) of **2** were a little similar to those of **1**, The differences found were the absence of the acetate group signals at *δ*_C_ 172.1 (C-16) and 21.3 (C-17) in compound **2**, other differences such as the NMR data in C-7, C-8, and C-9 suggested hydroxy group is linked to C-8 in compound **2**. The structure of **2** was further confirmed by the correlations of HMBC and ^1^H-^1^H COSY spectra as shown in Figure 1.

The relative configurations at C-6, C-7, and C-8 in **2** were deduced by a ROESY experiment (Figure 2). The *syn* configuration of H-7 and H-6 were determined as described in compound **1**, and the *β*-orientation of H-7 and H-6 were assigned by the correlations of H-7/CH_3_-13 and H-6/CH_3_-13. The *β*-orientation of H-8 was established by the correlations of H-8/CH_3_-13, and the *Z*- and *E*-configuration of Δ^4,5^ and Δ^1,10^ were determined same to **1** by the correlations H-5/H-3a, H-2(a, b)/CH_3_-15, and H-5/H-1. Therefore, compound **2** was established as 10-methyl-6,7-dimethylmethylene-8*α*-hydroxy-4-aldehyde-(4*Z*,10*E*))-dicyclodecadiene (Figure 3), and this compound was named heishuixiecaoline B.

Compound **3** was obtained as white amorphous powder, which gave a molecular formula of C_15_H_22_O_2_, as deduced by HRESIMS (*m*/*z* 257.1510 [M+Na]^+^, calc. for C_15_H_22_O_2_Na, 257.1517), indicating five degrees of unsaturation. The IR spectrum showed the presence of hydroxyl (3433 cm^−1^), *α*,*β*-unsaturated aldehyde (1682 cm^−1^), and double-bond (1631 cm^−1^) absorptions.

Compound **3** could be assigned a similar structure to **2** by comparison of their ^1^H- and ^13^C-NMR spectra (Table 1 and Table 2). The major differences observed were the absence of the trisubstituted C=C double bond between C-1 and C-10 present in **3** and the appearance of an exocyclic double bond between C-10 (*δ*_C_ 149.0) and C-15 (*δ*_C_ 113.4) and the oxygenated methine signal at *δ*_C_ 68.5 (C-1) in **3**. These were confirmed by the HMBC correlations from H-15 (*δ*_H_ 5.07, 5.12) to C-1, C-9 (*δ*_C_ 37.5), and C-10. The other correlations in the HMBC and ^1^H-^1^H COSY spectra as shown in Figure 1 confirmed the connectivities in compound **3**.

On the basis of the ROESY correlations (Figure 2), the relative configuration of **3** was determined to be the same as that of **2**, with H-1 assigned as *α*-oriented by the correlations of H-7/H-9a (*β*-H) and H-1/H-9b (*α*-H). The NOE correlations of H-3a/H-5 confirmed the *Z*-configuration of the double bond between C-4 and C-5. Thus, the structure of compound **3** (named heishuixiecaoline C) was assigned as 6,7-dimethylmethylene-4-aldehyde-1*β*-hydroxy-10(15)-ene-(4*Z*)-dicyclodecylene (Figure 3).

Known compounds were identified as volvalerenal C (**4**) [13], (+) pinoresinol-4,4'-di-*O*-*β*-D-glucopyranoside (**5**) [14], (+) pinoresinol-8-*O*-*β*-D-glucopyranoside (**6**) [15], 8-hydroxypinoresinol-4,4'-di-*O*-β-D-glucopyranoside (**7**) [16], (+) 8-hydroxypinoresinol-4'-*O*-*β*-D-glucopyranoside (**8**) [14], (+) pinoresinol-4-*O*-*β*-D-glucopyranoside (**9**) [17], (+) 8-hydroxypinoresinol (**10**) [14], (+) 8-hydroxypinoresinol-4-*O*-*β*-D-glucopyranoside (**11**) [14], patrinoside (**12**) [18], and kanokoside A (**13**) [19] by comparing their NMR spectroscopic data with the literature values. The structures of compounds 1-13 are shown in Figure 3.

The neuroprotective effects of compounds **1**–**13** against Aβ_25–35_ induced cell death in PC12 cells were assessed using an established MTT (3-(4,5-dimethylthiazol-2-yl)-2,5-diphenyl-2H-tetrazolium bromide) assay. Aβ_25–35_ induced cytotoxicity (56.94 ± 1.30% viability) in the cells when it was added at a concentration of 20 μM for 24 h. When PC12 cells were pre-incubated with vitamin E or compounds **1**–**15**, the toxicity of Aβ_25–35_ was significantly alleviated by vitamin E and compounds **1**–**7** in a dose-dependent manner (Table 3). While compounds **8**–**13** showed negligible protective effects on the cell viability (data not shown).

## 3. Experimental

### 3.1. General

The NMR spectra were recorded on a Bruker DPX 400 (400 MHz for ^1^H-NMR and 100 MHz for ^13^C-NMR, respectively, instrument (Bruker SpectroSpin, Karlsruhe, Germany). Chemical shifts are given as *δ* values with reference to tetramethylsilane (TMS) as an internal standard, and coupling constants are given in Hz. The HRESIMS analyses were conducted on Xero Q Tof MS spectrometer (Waters, Milford, MA, USA); Preparative HPLC was carried out on a Waters 600 instrument equipped with a Waters UV-2487 detector. A Waters Sunfire prep C18 OBD (19 × 250 mm i.d.) column was used for preparative purpose. IR Spectra (Shimadzu FTIR-8400S, Kyoto, Japan); Anal. TLC (silica gel 60 F254, Merck, Darmstadt, Germany). Column chromatography (CC): silica gel (200–300 mesh, Haiyang Chemical Group Co. Ltd, Qingdao, China); ODS-A (120A, 50 mm; YMC, Kyoto, Japan). Macroporous absorption resin (AB-8 Crosslinked Polystyrene, Nan Kai, Tianjin, China) was employed for column chromatography. PC12 cells obtained from Institute of biochemistry and cell biology (Shanghai, China) were grown in Dulbecco’s modified Eagle’s medium (DMEM) (Hyclone, NRH0020), supplemented with 5% fetal bovine serum and 1% antibiotic mixture comprising penicillin-streptomycin, in a humidified atmosphere at 37 °C with 5% CO_2_. Microplate reader (Safire2, Tecan Group Ltd., Maennedorf, Switzerland) was used to determine the cell viability.

### 3.2. Plant Material

The roots and rhizomes of *V. amurensis* were collected from the Great Xing’an Mountains area in 2010. The original plant was identified by Xiaowei Du of Heilongjiang University of Chinese Medicine. A voucher specimen (No. 20100806) was deposited at the Herbarium of Heilongjiang University of Chinese Medicine, China.

### 3.3. Extraction and Isolation

The dried roots and rhizomes of *V. amurensis* (7.0 kg) was extracted with 95% EtOH (56 L) for 2 h × 3 times under reflux conditions to give a residue (578.2 g) after removal of solvent under reduced pressure. The EtOH extract was suspended in H_2_O and then partitioned with petroleum ether (5 × 4 L). The remaining water extract (450.6 g) was fractioned by AB-8 macroporous resin column (10 × 60 cm) with H_2_O, 50% and 95% EtOH. The obtained 50% EtOH fraction (153.0 g) possesses potential therapeutic effect towards AD. The 50% EtOH fraction (100.0 g) was subjected to silica gel (200–300 mesh) column chromatography, eluted with CHCl_3_-CH_3_OH (from 50:1 to 1:1, v/v) to afford fractions I–VI. Fraction I (19.5 g) was subjected to column chromatography over silica gel, eluted with petroleum ether-EtOAc (from 40:1 to 1:1, v/v), to give six fractions, I_1_–I_6_. Fraction I_3_ (5.1 g) and I_4_ (3.4 g) was chromatographed over silica gel, eluted with petroleum ether-EtOAc (from 40:1 to 1:1, v/v), to afford fractions I_3_a-I_3_f and I_4_a–I_4_f. Compound **1** (25 mg) and **3** (32 mg) were isolated from fraction I_3_b by repeated column chromatography over silica gel, eluted with ether-EtOAc (from 40:1 to 15:1, v/v). Fraction I_4_a was subjected to column chromatography over silica gel eluted with petroleum ether-EtOAc (from 20:1 to 5:1, v/v) to obtain Compound **2** (35 mg).

*Heishuixiecaoline A* (**1**): white amorphous powder; ${\left[\alpha \right]}_{D}^{20}$ +93.9 (*c* 0.102, MeOH); UV (MeOH) *λ*_max_ (log*ε*) 257.5 nm; IR (KBr): 1736, 1682, 1625, 1460, 1450, 1373, 1244, 1139, 1015cm^−1^; ^1^H-NMR (CD_3_OD) and ^13^C-NMR (CD_3_OD) data are shown in Table 1 and Table 2; HRESIMS *m*/*z* 299.1619 [M+Na]^+^ (calcd for C_17_H_24_O_3_Na, 299.1623).

*Heishuixiecaoline B* (**2**): white amorphous powder; ${\left[\alpha \right]}_{D}^{20}$ +100.2 (*c* 0.104, MeOH); UV (MeOH) *λ*_max_ (log*ε*) 261 nm; IR (KBr): 3429, 2938, 2813, 2706, 1689, 1624, 1452, 1370, 1184, 1002 cm^−1^; ^1^H-NMR (CD_3_OD) and ^13^C-NMR (CD_3_OD) data are shown in Table 1 and Table 2; HRESIMS *m*/*z* 257.1514 [M+Na]^+^ (calcd for C_15_H_22_O_2_Na, 257.1517).

*Heishuixiecaoline C* (**3**): white amorphous powder; ${\left[\alpha \right]}_{D}^{20}$ −22.3 (*c* 0.112, MeOH); UV (MeOH) *λ*_max_ (log*ε*) 266.9 nm; IR (KBr): 3433, 1682, 1631, 1443, 1381, 1264, 1190 cm^−1^; ^1^H-NMR (CD_3_OD) and ^13^C-NMR (CD_3_OD) data are shown in Table 1 and Table 2; HRESIMS *m*/*z* 257.1510 [M+Na]^+^ (calcd for C_15_H_22_O_2_Na, 257.1517).

### 3.4. Determination of Cell Viability

Cell viability was measured by quantitative colorimetric assay with 3-(4,5-dimethylthiazol-2-yl)-2,5-diphenyltetrazolium bromide (MTT) method as described previously [20]. Briefly, the cells were cultured at a density 5 × 10^4^ cells per well in growth medium for 24 h in 96-well plates, and then preincubated without or with various concentrations (5, 12, 25 μM) of compounds **1**–**13**, which was followed 24 h later by exposure to 20 μM aggregated Aβ_25–35_ (Sigma, St. Louis, MO, USA) prepared as described previously [21]. Vitamin E was used as a reference compound [22]. 25 μL/well of MTT solution (5 mg/mL) was added and cells were incubated at 37 °C for 4h. Supernatants were then aspirated off and formazan crystals were dissolved with DMSO. The optical density of each well was determined at 490 nm using a microplate reader. Results were expressed as the percentages of reduced MTT, assuming the absorbance of control cells as 100%. For comparing the results of MTT between the study groups, the *t*-test was used.

## 4. Conclusions

We investigated the chemical constituents of *V. amurensis* based on its activity towards AD for the first time and three new germacrane-type sesquiterpenoids were obtained. Their structures were identified as 10-methyl-6,7-dimethylmethylene-8*β*-acetoxy-4-aldehyde-(4*Z*,10*E*)-dicyclodecadiene (**1**), 10-methyl-6,7-dimethylmethylene-8*α*-hydroxy-4-aldehyde-(4*Z*,10*E*))-dicyclodecadiene (**2**), and 6,7-dimethyl-methylene-4-aldehyde-1*β*-hydroxy-10(15)-ene-(4*Z*)-dicyclodecylene (**3**), respectively. Seven known cpompounds were also isolated and identified. All these compounds were screened and cell tests showed that germacrane-type sesquiterpenoids **1**–**4** and lignans **5**–**7** were the active components of *Valeriana amurensis* against AD.

## Figures and Tables

**Table 1:** The ^1^H-NMR data of **1**–**3** in CD_3_OD (*δ* in ppm, recorded at 400 MHz).

No.	1	2	3
1	5.31 (1H, dd, 4.4, 9.6)	5.25 (1H, dd, 4.0, 9.2)	3.59 (1H, dd, 6.8, 9.2)
2a	2.09 (1H, m)	2.04 (1H, m)	1.97 (2H, m)
2b	2.15 (1H, m)	2.11 (1H, m)	
3a	2.09 (1H, m)	2.03 (1H, m)	1.78 (1H, m)
3b	2.69 (1H, m)	2.69 (1H, dd, 4.0, 11.2)	2.45 (1H, m)
5	6.57 (1H, d, 9.8)	6.56 (1H, d, 9.6)	6.49 (1H, d, 6.8)
6	1.85 (1H, t, 9.8)	1.73 (1H, t, 9.6)	1.46 (1H, dd, 6.8, 11.2)
7	1.41 (1H, t, 9.8, 10.8)	1.17 (1H, t, 10.8)	0.86 (1H, dt, 2.4, 12.4)
8	4.50 (1H, dt, 3.2, 10.8)	3.35 (1H, dt, 4.4, 10.8)	1.78 (1H, ddd, 4.0, 4.0, 14.4); 1.04 (1H, m)
9	2.20 (1H, dd, 2.8, 11.2); 2.30 (1H, t, 11.2)	2.23 (2H, m)	2.13(1H, dt, 4.8, 12.8); 2.49(1H,m)
12	1.18 (3H, s)	1.19 (3H, s)	1.12 (3H, s)
13	1.20 (3H, s)	1.34 (3H, s)	1.14 (3H, s)
14	9.30 (1H, s)	9.24 (1H, s)	9.35 (1H, s)
15	1.34 (3H, s)	1.28 (3H, s)	5.07(1H, brs); 5.12(1H, brs)
17	2.03 (3H, s)	--	--

**Table 2:** The ^13^C-NMR and DEPT data of **1**–**3** in CD_3_OD (*δ* in ppm, recorded at 100 MHz).

No.	1	2	3
1	128.7 (CH)	127.7 (CH)	68.5 (CH)
2	28.3 (CH_2_)	28.3 (CH_2_)	30.1 (CH_2_)
3	24.5 (CH_2_)	24.4 (CH_2_)	22.8 (CH_2_)
4	145.1 (C)	144.1 (C)	146.3 (C)
5	155.8 (CH)	157.4 (CH)	155.4 (CH)
6	31.9 (CH)	32.1 (CH)	28.5 (CH)
7	40.8 (CH)	44.1 (CH)	36.3 (CH)
8	73.6 (CH)	69.9 (CH)	23.2 (CH_2_)
9	47.2 (CH_2_)	50.8 (CH_2_)	37.5 (CH_2_)
10	133.7 (C)	134.6 (C)	149.0 (C)
11	23.7 (C)	23.5 (C)	21.8 (C)
12	28.3 (CH_3_)	28.7 (CH_3_)	28.1 (CH_3_)
13	16.0 (CH_3_)	16.0 (CH_3_)	16.2 (CH_3_)
14	196.6 (CH)	196.6 (CH)	196.7 (CH)
15	18.2 (CH_3_)	18.5 (CH_3_)	113.4 (CH_2_)
16	172.1 (C)	--	--
17	21.3 (CH_3_)	--	--

**Table 3:** Neuroprotective effects of Compounds **1**–**7** against Aβ_25–35_-induced PC12 cells death.

Compound	Cell viability (%)		
	5 μM	** 12 μM	* 25 μM
1	64.43 ± 3.02	69.77 ± 2.45	77.24 ± 2.14
2	65.16 ± 4.20	70.31 ± 3.38	78.33 ± 3.29
3	65.07 ± 3.26	72.97 ± 3.47	77.84 ± 2.18
4	64.85 ± 4.14	70.83 ± 3.12	80.38 ± 4.46
5	68.59 ± 2.63	75.81 ± 4.79	84.75 ± 2.66
6	71.52 ± 3.34	78.78 ± 4.22	89.54 ± 3.27
7	67.79 ± 2.26	75.80 ± 2.19	85.04 ± 3.23
Vitamin E	61.76 ± 1.48	70.47 ± 2.56	79.80 ± 2.72

Effects of the tested compounds on Aβ-induced PC12 cell death. Cell viability was measured by MTT assay. Results are expressed as mean ± SD (n = 8) of three independent experiments. The 100% value was obtained from untreated control cells. * Significant difference compared 25 μM with 5 and 12 μM of compounds **1**–**7** (* *p* < 0.01), respectively. ** Significant difference compared 12 μM with 5 of compounds **1**–**7** (** *p* < 0.05), respectively.
